# DNA supercoiling and transcription in bacteria: a two-way street

**DOI:** 10.1186/s12860-019-0211-6

**Published:** 2019-07-18

**Authors:** Charles J. Dorman

**Affiliations:** Department of Microbiology, Moyne Institute of Preventive Medicine, Trinity College Dublin, Dublin 2, Ireland

## Abstract

**Background:**

The processes of DNA supercoiling and transcription are interdependent because the movement of a transcription elongation complex simultaneously induces under- and overwinding of the DNA duplex and because the initiation, elongation and termination steps of transcription are all sensitive to the topological state of the DNA.

**Results:**

Policing of the local and global supercoiling of DNA by topoisomerases helps to sustain the major DNA-based transactions by eliminating barriers to the movement of transcription complexes and replisomes. Recent data from whole-genome and single-molecule studies have provided new insights into how interactions between transcription and the supercoiling of DNA influence the architecture of the chromosome and how they create cell-to-cell diversity at the level of gene expression through transcription bursting.

**Conclusions:**

These insights into fundamental molecular processes reveal mechanisms by which bacteria can prevail in unpredictable and often hostile environments by becoming unpredictable themselves.

## Background

Variable DNA supercoiling is a fundamental principle in the control of gene expression in bacteria [1–4]. DNA is usually negatively supercoiled in bacterial cells because it contains a deficit of helical turns [5–7]. In its B form, the strands of the DNA duplex make one complete turn every 10.5 base pairs. Increasing the frequency of turning tightens the duplex and results in positive writhing as the axis of the double helix coils around itself in search of a minimal energy conformation. Removing turns through underwinding the duplex has the opposite effect, causing the duplex to writhe negatively. If the DNA is neither under- nor overwound, it adopts a relaxed conformation [8].

**Fig. 1:**
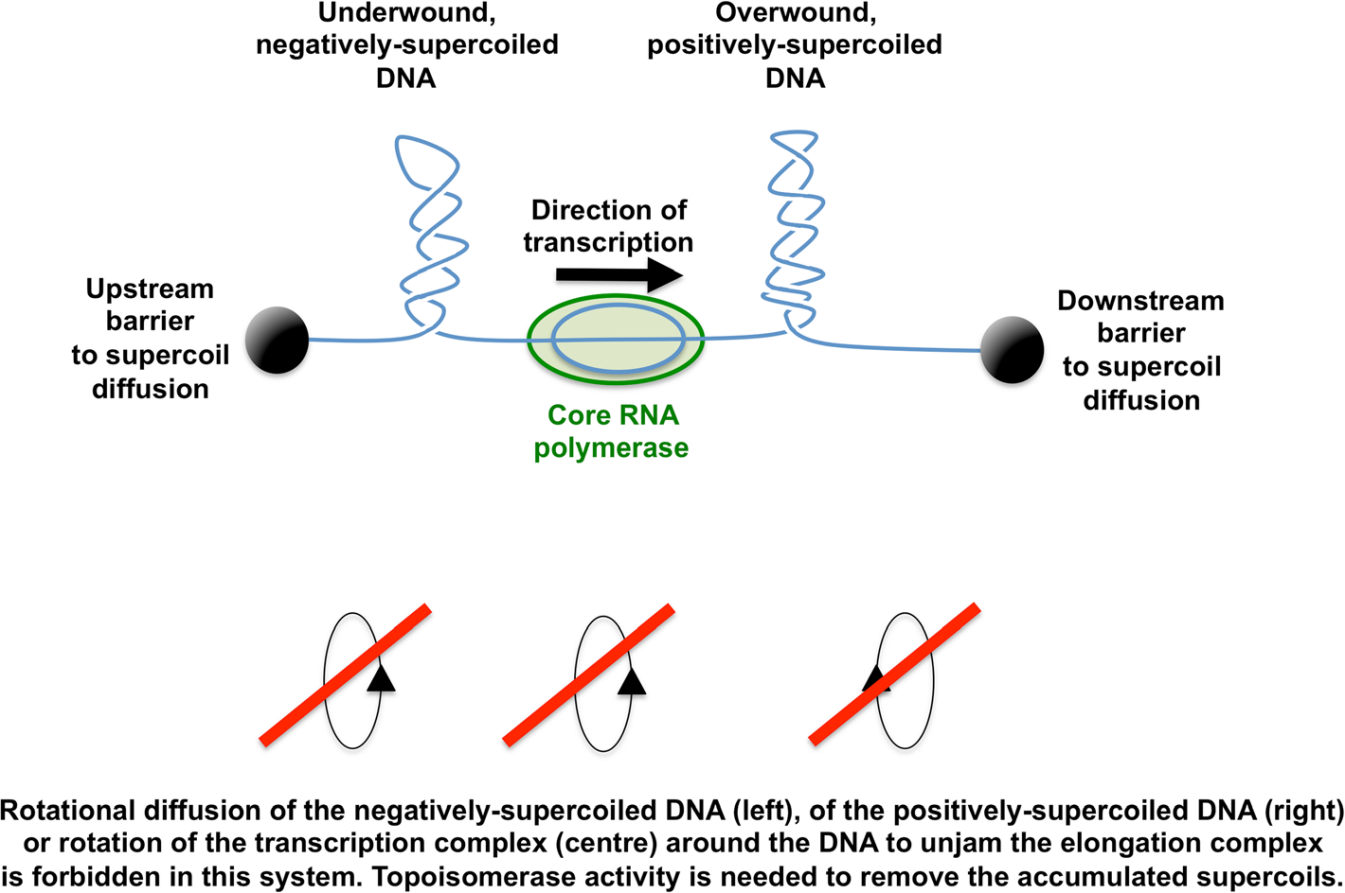
Twin supercoiling domain model. This is the model proposed by Liu and Wang (1987) and supported by numerous independent experiments. Core RNA polymerase is engaged in transcript elongation: mRNA, ribosomes and nascent polypeptide are omitted for clarity. As the coupled transcription-translation complex moves from left to right, the DNA template ahead becomes over wound (positively supercoiled plectonemes) while the DNA behind becomes under wound (negatively supercoiled plectonemes). This situation will halt transcription as the machinery jams because: (**a**) the domains of supercoiled DNA cannot be removed by supercoil diffusion due to the presence of topological barriers (black spheres at the ends of the DNA) and (**b**) the bulky transcription-translation complex cannot rotate around the DNA to relieve the torsional tension in the duplex DNA. Instead, DNA gyrase will remove the positive supercoils while the negative ones are relaxed by DNA topoisomerases I and/or IV. Interference with these relaxation processes can result in undesirable outcomes, such as R-loop formation (Fig. 2). Topological barriers can arise due to head-to-head transcription complex collisions and by collisions between converging replisomes and transcription complexes; they can be produced by nucleoprotein complexes and by distortions (e.g. sharp bends) in the DNA duplex. The oval arrows at the bottom of the figure represent possible rotational solutions to these topological problems: each of these solutions is ruled out (red lines) because rotation of the DNA and/or the transcription complex cannot occur, for the reasons summarised in (**a**) and (**b**) above

Underwound DNA experiences torsional stress that is usually neutralised by wrapping the DNA around proteins to constrain supercoils, by allowing the DNA duplex to writhe and/or by allowing some of the pairs of hydrogen-bonded bases to unpair [8]. In living bacteria, all three solutions are employed. DNA binds a variety of proteins that can constraint supercoils and nucleoid-associated proteins have a special role in providing this function [9]. At any given time in the growth cycle, about 40% of the DNA in the bacterial genome is free of protein and can participate in supercoiling through the formation of DNA plectonemes, segments of interwound, or braided, double-stranded DNA (Fig. 1) [10]. The breakage of hydrogen bonds between pairs of bases due to torsional stress assists such processes as transcription, where the generation of single-stranded bubbles in the double-stranded DNA is essential. This requirement provides one mechanistic link between transcription and DNA supercoiling.

Another link arises from the ability of DNA topology to influence the presentation of promoters and associated binding sites for transcription regulatory proteins to RNA polymerase and transcription factors, respectively. Writhing and looping of the DNA can bring sites along the molecule closer together to enhance or to inhibit the formation of closed transcription complexes or their isomerization to open complexes [11].

**Fig. 2:**
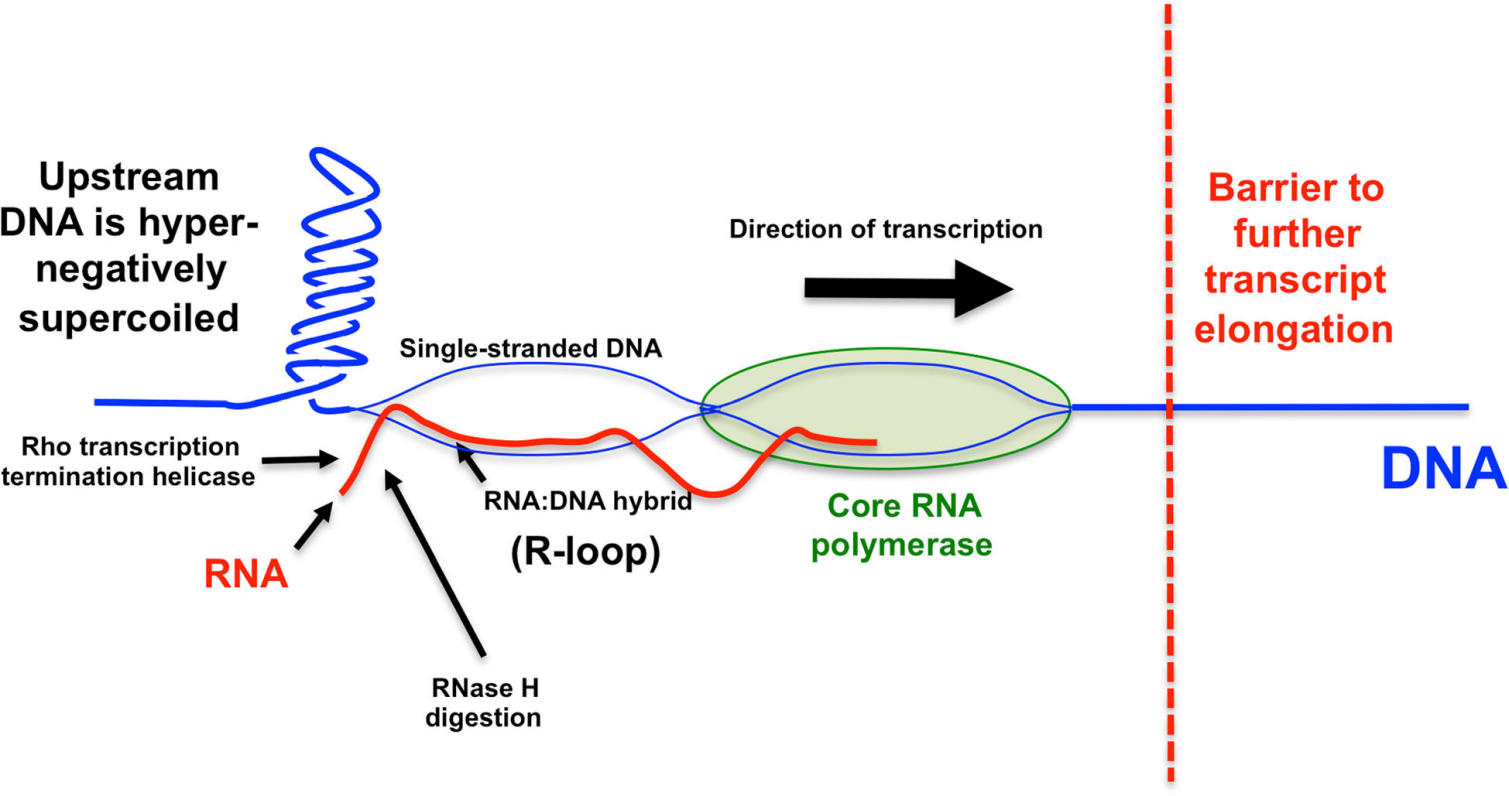
DNA negative supercoiling and R-loop formation during transcription. When RNA polymerase (green) reads a G + C-rich DNA template, stalls and backtracks, it leaves a domain of hyper-negatively supercoiled behind. The associated stalling of transcription may allow the RNA transcript (red) to base pair with its DNA template strand (blue), leaving the non-transcribed strand as a single-stranded bubble. Other impediments to RNA polymerase progression include head-on collisions with other transcription units or with replisomes (the barrier is represented by the red vertical dotted line). Loss of the DNA relaxing activity of topoisomerase I promotes R-loop formation because it encourages the accumulation of hyper-negative-superhelicity in DNA that is being transcribed (or replicated). Failure to process and remove RNA loops can lead to DNA damage, including double-stranded breaks and hyper-recombination. RNase H eliminates R-loops by removing the RNA component of the RNA:DNA hybrid in the R-loop. The Rho transcription terminating helicase can suppress R-loop formation by preventing RNA polymerase backtracking

**Fig. 3:**
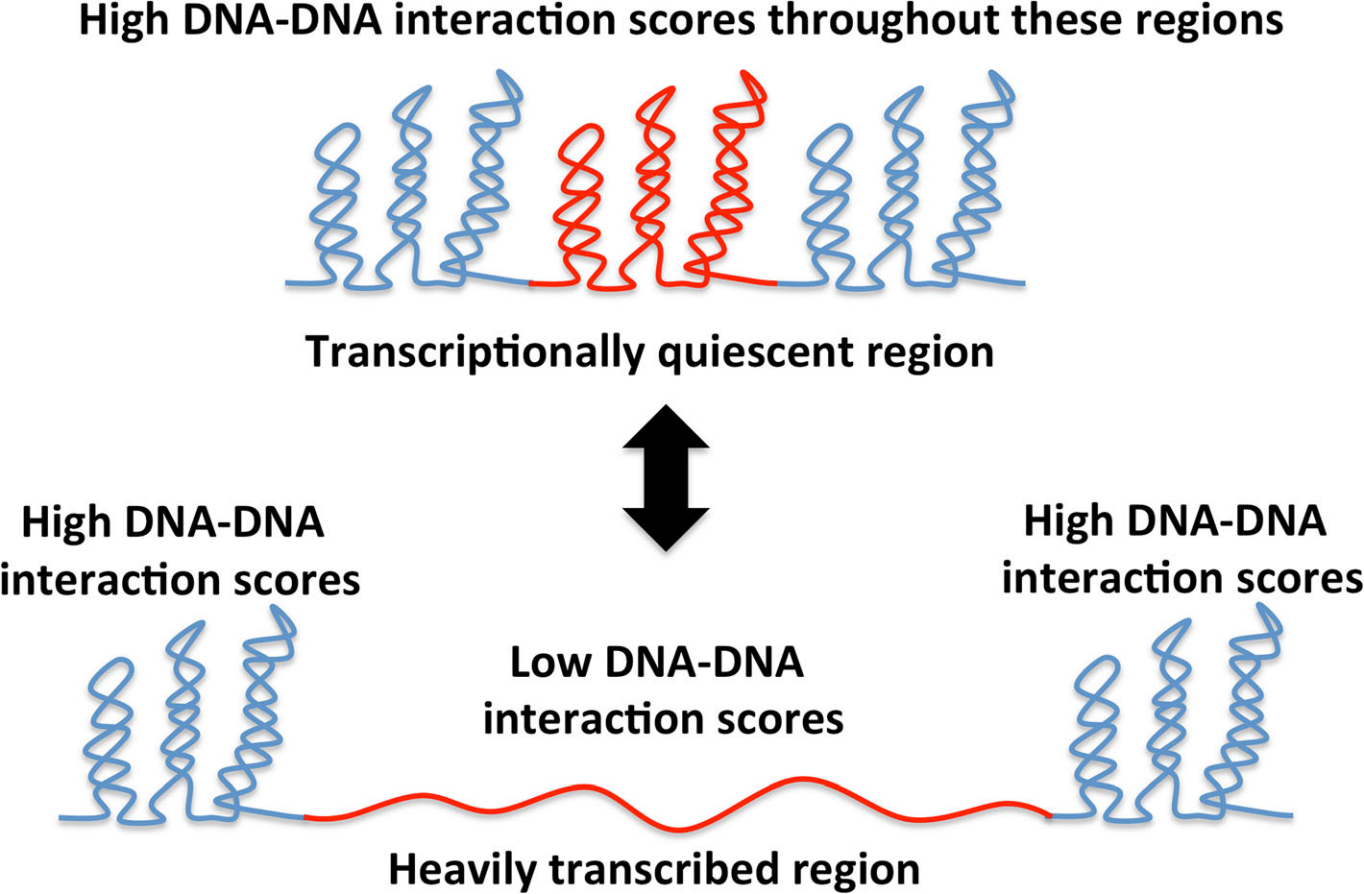
Transcription DNA, supercoiling and chromosome architecture. Data from chromosome conformation capture experiments indicate that long, heavily-transcribed transcription units can form barriers to DNA-DNA interaction [16]. The transcribed region (red) has few plectonemes and insulates the flanking regions that are rich in plectonemically interwound DNA. Cessation of transcription in the red zone allows plectonemic wrapping of DNA to be restored, re-establishing DNA-DNA contacts and allowing interactions between the red zone and the flanking regions. Activating and inhibiting transcription in the red region lowers and raises, respectively, the barrier that insulates it from its neighbouring genomic regions. The insulating mechanism does not involve influencing supercoil diffusion, nor is it dependent on translation of the transcripts within the heavily transcribed region

Forming an open transcription complex involves the generation of a single-stranded bubble in the DNA, a process that removes local DNA twist. As transcription elongation gets underway, the DNA downstream of the transcription complex becomes overwound while that in the upstream zone becomes underwound (Fig. 1). These events show that the act of transcription elongation is a generator of DNA supercoiling [12–14]. Eliminating the supercoils is essential if elongation is to continue, otherwise the transcription complex will jam, the nascent transcript will form base-pairs with its DNA template to produce an R-loop, leading to RNA polymerase stalling or even backtracking before the transcription termination site at the end of the gene can be reached (Fig. 2) [15]. The influence of transcription elongation on local DNA topology is exacerbated when very long transcripts are generated, as is the case with operons encoding ribosomal RNA and other components of the translation apparatus. In these cases, the impact on chromosome architecture becomes detectable with chromosome conformation capture methods (Fig. 3) [16].

The application of single-molecule methods has deepened our understanding of cell-to-cell variation in processes such as transcription. Not every cell in a population of genetically identical bacteria will respond to a transcription-inducing signal. Instead, the transcriptional response to the signal will occur in just a subset of the bacteria. A role for transcription-dependent local positive supercoiling of the DNA in generating this cell-to-cell variety has been proposed [17, 18], further emphasising the intimacy and mutuality of the relationship between transcription and DNA topology in bacteria.

### Topoisomerases manage local DNA topology

Tight links between transcription elongation complexes and topoisomerases were predicted on theoretical grounds over 30 yrs ago [19] and confirmed experimentally [20–26]. DNA gyrase is a type II topoisomerase that uses ATP hydrolysis to introduce negative supercoils into relaxed DNA; it uses the same ATP-dependent type II mechanism to eliminate positive supercoils [27–30] and an ATP-independent mechanism to relax negative supercoils [29, 31, 32]. Gyrase accompanies RNA polymerase and manages the topological state of the downstream DNA during transcription elongation so that the process can continue to termination [33]. About 2,000 copies of RNA polymerase are engaged in transcription in the model organism *Escherichia coli* during periods of maximum growth [34]. Each RNA polymerase introduces about 6 positive supercoils per second [35] and 300 gyrase molecules are devoted to their elimination [36]. The negatively supercoiled domain that is generated upstream of the transcription elongation complex is processed by topoisomerases that can relax underwound DNA [33]. These principles also apply in the case of the moving replisomes as they copy the chromosome [37, 38]. Type II topoisomerases help to resolve the DNA topological conflicts arising from head-on transcription-replication collisions [39]. These conflicts create a potentially dangerous scenario for the cell because their resolution by type II enzymes involves the transient generation of double-stranded breaks in DNA [40]. If these breaks are not resealed efficiently following the passage of the DNA duplex through the double-stranded gap, permanent DNA damage may result [39, 41, 42]. Head-on collisions between elongating transcription complexes, or between such a complex and a replisome, can lead to R-loop formation in the underwound zone upstream of the transcription complex. Processing of the RNA and the relaxation of the negatively supercoiled DNA are needed to eliminate the R-loop and to restart the stalled transcription complex [43–45].

### DNA supercoiling and transcription initiation

Surveys have been conducted to detect bacterial genes that exhibit changes in expression that correlate with changes in DNA supercoiling [46, 47]. These changes in DNA supercoiling are brought about using drugs that inhibit the activity of a subset of the DNA gyrase molecules in the cell (gyrase is an essential enzyme, so complete removal of its activity is lethal) or using mutants lacking a non-essential topoisomerase (e.g. topoisomerase I) or that express a topoisomerase with diminished activity. Some genes show enhanced expression when DNA is relaxed while others exhibit higher levels of expression when DNA is negatively supercoiled [47, 48]. A requirement for negative supercoiling is intuitively appealing because it is consistent with the need to disrupt base pairing to form an open transcription complex. A role for DNA relaxation in directly promoting gene expression is more difficult to rationalise. It may arise when changes to DNA twist alter the presentation of binding sites to regulatory proteins that rely on indirect readout (DNA shape) to bind DNA. Many proteins in this class (e.g. LysR-type transcription regulators) use winged-helix-turn-helix binding motifs that engage both the major and minor grooves in DNA [49]. DNA relaxation may facilitate the binding [50], with the effect being especially strong at sites in A + T-rich DNA where the minor groove is at its narrowest [51].

### DNA supercoiling and transcription elongation/termination

Following the isomerization of the closed transcription complex to an open one, an RNA transcript is rapidly synthesized within a ternary elongation complex consisting of the core RNA polymerase, the DNA template and the nascent transcript [52, 53]. A short section of melted DNA of about 10 to 12 nucleotides makes up the transcription bubble. The newly transcribed RNA and the DNA template remain base paired over about 10 nucleotides within the complex and two ‘zip locks’, one upstream and one downstream of the complex sustain the conformation of the RNA:DNA hybrid [54, 55]. The translocation of the elongation complex overwinds the DNA ahead and underwinds the DNA behind (Fig. 1) and several factors can intervene to cause the complex to pause/terminate, and even to backtrack (Fig. 2). The NusA protein enhances pausing and the NusG protein counteracts this effect [56]. UvrD promotes DNA repair by promoting backtracking to reveal patches of UV-damaged DNA [57]. Nascent RNA extruding from the backtracked transcription complex are cleaved via a mechanism that is induced by the GreA and GreB proteins, restoring the 3′ end of the transcript at the active center of the transcription complex [58, 59]. Because transcription and translation are coupled, a failure to load ribosomes allows the Rho transcription termination factor to bind to the transcript and cause pausing/termination and, possibly, backtracking (Fig. 2) [60]. The negative supercoiling of the DNA upstream of the paused/backtracked transcription complex creates and opportunity for R-loop formation. These RNA:DNA hybrids are removed by the DNA relaxing activity of topoisomerase I and the RNA degrading activity of RNase H (Fig. 2) [43–45].

### DNA supercoiling homeostasis

The promoters of the genes that encode DNA gyrase are more active when the DNA is relaxed [61–65]. The FIS nucleoid-associated protein represses gyrase gene transcription and the *fis* gene has a promoter that is stimulated by negative supercoiling [66]. In contrast, transcription of the *topA* gene, encoding the DNA-relaxing topoisomerase I, is stimulated by negative DNA supercoiling [67, 68]. These topoisomerase genes, with their opposite preferences for DNA supercoiling, form the basis of a system for the homeostatic management of global DNA supercoiling levels in the cell, presumably maintaining the supercoiled state of DNA within limits that are appropriate for cell survival [2, 33, 62, 69–74]. The mechanisms are consistent with the predictions made in the twin supercoiling domain model [19] (Fig. 1): topoisomerases are recruited principally to the most active transcription units with the binding of gyrase and topoisomerase I being to the expected locations in divergently- and convergently-transcribed genes, with the topoisomerases adopting the expected locations upstream and downstream of RNA polymerase in vivo [67]. When topoisomerase I is recruited to promoters, it becomes active in response to the negative supercoiling induced by transcription elongation [75].

### Physiology, stress and DNA supercoiling

A correlation has been reported between DNA supercoiling and the growth cycle of model bacteria. In essence, negative supercoiling of DNA correlates with periods of high metabolic flux, with bacteria in the exponential phase of growth having the most negatively supercoiled DNA and those in the lag and stationary phases having DNA that is relaxed [76, 77]. The stress-and-stationary-phase sigma factor, RpoS, accumulates in bacteria that experience growth arrests from multiple causes [78]. Unlike the housekeeping sigma factor RpoD, RpoS initiates transcription efficiently from promoters in relaxed DNA templates, and is inhibited by negatively supercoiled DNA until the appropriate level of DNA relaxation is achieved [79].

DNA supercoiling has also been implicated in the operation of the stringent response, an event that is triggered by multiple causes in different bacterial species, and one of whose key roles is to inhibit the production of the translation machinery of the cell [80–85]. In *E. coli,* promoters that are subject to negative control by the stringent response have a G + C-rich discriminator sequence between the + 1 and − 10 promoter elements [86–90] that makes contact with Conserved Region 1.2 in the RNA polymerase housekeeping sigma factor, RpoD [91]. It has been suggested that because this feature lies in the part of the promoter that must become single-stranded in open complex formation, it inhibits transcription initiation in the relaxed DNA templates that obtain in nutritionally stressed bacteria [66, 92]. Promoters that are stimulated during the stringent response have an A + T-rich discriminator [93].

Environmental stresses correlate with changes to DNA supercoiling levels. For example, growing *E. coli* on glucose, the organism’s preferred carbon source, is associated with DNA that is more negatively supercoiled whereas DNA relaxation is correlated with growth on poorer carbon sources [94]. Glucose uptake and metabolism produce a higher ration of ATP to ADP and the [ATP]/[ADP] ratio influences the activity of the ATP-dependent DNA supercoiling activity of gyrase [74, 95–98]. However, experiments with dinitrophenol, an uncoupler of the cytoplasmic-membrane-based respiratory chain, failed to correlate ATP synthesis and negative supercoiling of reporter plasmids [94]. Perhaps targeting ATP synthesis by the membrane-located F_0_F_1_ ATPase caused the contribution of adenylate kinase to ATP production to be overlooked: exposing *E. coli* to osmotic upshock, a stress that removes water from the cytoplasm, is accompanied by a high demand for ATP that is met, in part, by adenylate kinase [99]. In the initial stages of upshock, reporter plasmids in *E. coli* and *Salmonella* become more negatively supercoiled [96, 100].

Supercoiling shifts have also been reported following, inter alia*,* acid stress [101–104], intracellular growth [102, 105], osmotic stress [46, 79, 96, 100, 106–109], oxidative stress [110], changes to oxygen levels [95, 111–117] and thermal stress [118]. These observations suggest that alterations to the chemical or physical composition of the environment and the subsequent effects on metabolism can produce a shift in the superhelicity of the genetic material of a bacterium [1]. Is this shift utilised at the level of the transcriptional response to the environmental changes? In many cases, it is. For example, genes involved in transporting into the cell compatible solutes to replace the water lost in osmotic upshock are transcriptionally activated by negative supercoiling of DNA [100] while genes that respond to acid stress have promoters that are triggered when DNA relaxes, and DNA relaxation is a feature of bacteria that are shifted to low pH [102, 103].

### Transcription, DNA supercoiling and chromosomal architecture

DNA supercoiling and transcription are mutually influential, lending themselves to the analogy of a two-way street. They also influence the architecture of their street: the bacterial chromosome. Long, heavily transcribed genetic units establish contact barriers between flanking regions of the chromosome (Fig. 3). These barriers are maintained by RNA polymerase traffic and the associated disturbance to local DNA supercoiling [16]. Single-molecule studies have revealed that the plectonemic (braided, or interwound) form of supercoiled DNA is ‘pinned’ by A + T-rich DNA sequences found upstream of promoters that take up station at the apex of the plectoneme [119]. This finding offers the possibility of using bioinformatic methods to predict the positions of chromosome architectural features that are associated with DNA supercoiling.

Binding sites for DNA topoisomerase I and for DNA gyrase have been mapped in *Mycobacterium tuberculosis* by ChIP-Seq and found to occur at transcription units where they eliminate negative and positive supercoils, respectively [120]. DNA gyrase binding sites have been mapped by ChIP-chip [121] and gyrase-binding-and-DNA-cleavage sites have been mapped by ChIP-Seq in *E. coli* [122]. These cleavage sites are enriched downstream of heavily-transcribed genes, in keeping with the need to station the topoisomerase there to eliminate the positive supercoils generated by transcription elongation. Inhibition of transcription with rifampicin causes gyrase to be redistributed, again consistent with a tight link between transcription-generated positive supercoils and the presence of DNA gyrase [122].

Gyrase sites were not detected frequently in those parts of the *E. coli* chromosome that are known to be bound by the H-NS nucleoid-associated protein, a silencer of transcription. However, the activities of H-NS and gyrase do overlap, as has been shown in the transcriptional control of the *proU* operons of *E. coli* and *Salmonella* and other osmotically sensitive genes in bacteria undergoing osmotic stress [76, 100]. In addition, super-resolution imaging has shown that the H-NS protein becomes detached and excluded from the nucleoids of bacteria experiencing osmotic upshock in the stationary phase of the growth cycle; in exponential phase cells, this detachment and exclusion phenomenon is only seen for H-NS in the presence of the DNA gyrase inhibitor coumermycin [123]. This relationship provides a useful example of the connection between DNA gyrase and nucleoid associated proteins in bacteria. The NAP HU often acts as a partner to gyrase at the ends of heavily transcribed genes where the two proteins form a complex at repetitive palindromic DNA sequences [124, 125]. The integration host factor (IHF) NAP is also found at related sites [126, 127]. IHF and gyrase-mediated DNA supercoiling cooperate in recruiting proto-spacer sequences at CRISPR array leader sequences to extend the range of invading mobile genetic elements that can be detected and destroyed by this bacterial acquired immunity system [128, 129]. DNA binding proteins, including IHF and LRP, cooperate with DNA supercoiling to set and to reset the genetic switch that is responsible for the phase-variable expression of type 1 fimbriae and the choice between a planktonic and an attached, biofilm-associated lifestyle in *E. coli* [130–132].

### DNA supercoiling and transcriptional unpredictability

Transcription of highly expressed genes occurs in bursts [17, 133] and a role for local DNA supercoiling has been proposed in generating these stochastic events. Using single-molecule methods, Chong and colleagues found that, as positive supercoils build up ahead of the transcription elongation complex during the transcription of a highly-expressed gene, they first slow elongation before eventually feeding back onto the promoter where they inhibit transcription initiation [18]. The relaxation of the positive supercoils by DNA gyrase is necessary before transcription of the gene can resume. These processes, and the various actors required to operate them, produce opportunities for differential rates of gene expression among the genetically identical copies of this gene in each cell in the bacterial population. Different copies will be at different stages of the transcription cycle and will have individual numbers of positive supercoils, leading to different levels of transcription inhibition. Release from transcription inhibition by the relaxation of positive DNA supercoiling relies on the availability of DNA gyrase and ATP. The replication cycles of the chromosomes in the bacteria will not be synchronised, so the gene copy number will vary from cell to cell, generating further opportunities for variation in the expression of the gene. The relationship between mRNAs and their protein products is randomised at cell division, exacerbating the effect of stochastic events in gene expression in recently divided cells [134]. The expression of neighbouring genes represents yet-another cell-to-cell variable, as does the timing of the passage of the replication fork with the associated resetting of local protein binding patterns. These and other stochastic events introduce unpredictability into the expression of a specific gene, even when the gene is receiving signals that would be expected to activate its expression. The result is physiological variety at a population level and this can benefit the population through the generation of members with different degrees of competitive fitness. It may increase the likelihood that, in an unpredictable and dynamic environment, at least some members of the bacterial population will be prepared if novel environmental circumstances arise.

## Conclusions

The intimate connections between transcription and DNA supercoiling have been known for several decades, yet they remain something of a specialist interest in the research community. These processes will have to be appreciated more widely and in greater depth if a truly complete understanding of bacterial cell biology is to be achieved. It will be difficult to make progress efficiently in areas such as synthetic biology if our knowledge of the rules governing the operation of natural model organisms’ DNA-based transactions is incomplete. Similarly, understanding how bacteria exploit the DNA-supercoiling-transcription connection to drive cell-to-cell physiological diversity is important in the field of infectious disease, where such processes can aid in the emergence of outliers that differ from ‘the crowd’ in their competitive fitness. This last point is especially important in the light of our reliance on DNA-gyrase-inhibiting drugs to treat infection.

## Data Availability

This review article does not contain new data. Inquiries regarding the content of the paper should be sent to the author (cjdorman@tcd.ie).

## Abbreviations

ChIP: chromatin immunoprecipitation
NAP: nucleoid-associated protein;
